# ﻿*Glaridoglanis verruciloba* sp. nov., a new glyptosternine catfish (Siluriformes, Sisoridae) from the Zayul River in southeastern Tibet, China

**DOI:** 10.3897/zookeys.1262.172819

**Published:** 2025-12-10

**Authors:** Zheng Gong, Huanshan Wang, Yanchao Liu, Jianchuan Li

**Affiliations:** 1 College of Life Sciences, Zaozhuang University, Zaozhuang 277160, China Zaozhuang University Zaozhuang China; 2 Institute of Hydrobiology, Chinese Academy of Sciences, Wuhan 430072, China Chinese Academy of Sciences Wuhan China; 3 Key Lab of Biological Resources and Biosecurity of Xizang Autonomous Region, Institute of Plateau Biology of Xizang Autonomous Region, Lhasa 850030, China Institute of Plateau Biology of Xizang Autonomous Region Lhasa China; 4 Xizang Museum of Natural Science, Lhasa 850011, China Xizang Museum of Natural Science Lhasa China

**Keywords:** Cytochrome b gene, Glyptosterninae, morphological comparison, phylogenetic analysis, taxonomy

## Abstract

*Glaridoglanis verruciloba***sp. nov.** is described from the Zayul River in southeastern Tibet, China. It has long been misidentified as *G. andersonii*. This new species is diagnosed by the following combination of characters: an enlarged lower lip bearing 4–7 verruciform lobes on the central-posterior margin; an anus situated close to the origin of the anal fin; the ventral surface at the base of maxillary barbels densely covered with striae; 11 branched pectoral-fin rays; 5–6 branched anal-fin rays; and relatively short mandibular barbels. Molecular phylogenetic analyses of mitochondrial cytochrome b sequences further supported the validity of this new species, which is within a well-supported clade with substantial genetic divergence from *G. andersonii*.

## ﻿Introduction

The glyptosternine catfish (Siluriformes, Sisoridae) comprises 13 valid genera, including *Barbeuchiloglanis* Li, Dao & Zhou, 2022, *Chimarrichthys* Sauvage, 1874, *Creteuchiloglanis* Zhou, Li & Thomson, 2011, *Exostoma* Blyth, 1860, *Glaridoglanis* Norman, 1925, *Glyptosternon* McClelland, 1842, *Myersglanis* Hora & Silas, 1952, *Oreoglanis* Smith, 1933, *Parachiloglanis* Wu, He & Chu, 1981, *Pareuchiloglanis* Pellegrin, 1936, *Pseudecheneis* Blyth, 1860, *Pseudexostoma* Chu, 1979, and *Tremeuchiloglanis* Li, Dao & Zhou, 2022 (Fricke et al. 2025). Species of this group are morphologically distinctive, characterized by the extremely depressed head and body, highly modified mouth structure, and horizontally expanded pectoral and pelvic fins, which enable them to adhere to rocky substrates in fast-flowing mountain streams (Thoni and Gurung 2018). The distribution of glyptosternine catfishes is concentrated in the East Himalayas, Indo-Burman Ranges, Gaoligong Mountains, Shan Hills and Kachin Hills; these catfishes range from Uzbekistan in the west to South China in the east (Sheraliev and Peng 2021; Chen et al. 2025). Phylogenetic studies based on mitochondrial and nuclear markers suggest that this group diverged from other sisorid catfishes during the late Miocene, a period coinciding with the major phases of Himalayan uplift (Yu and He 2012).

The genus *Glaridoglanis*, which is endemic to the Irrawaddy and the Yarlung Tsangpo–Brahmaputra drainages, currently contains two valid species: *G. andersonii* (Day, 1870) and *G. ramosa* Ng & Kottelat, 2022 (Ng and Kottelat 2022). *Glaridoglanis* species exhibit typical torrent-catfish morphology of the subfamily Glyptosterninae, including the dorsoventrally flattened body, greatly enlarged paired fins with horizontally inserted pinnate first rays, and an inferior mouth with well-developed suctorial lips (Thoni and Gurung 2018). They are further distinguished by the robust, chisel-shaped dentition on both jaws, which is adapted to exploit the primary food resources of these fishes (Chu 1979). Ecologically, these species inhabit cold, oxygen-rich torrents with rocky substrates, where they use their modified pectoral and pelvic fins for adhesion and movement against strong currents (Wu and Wu 1991).

Historically, *G. andersonii* was the only species of *Glaridoglanis* known from the Zayul (= Chayu) River (Chu and Mo 1999; Ng and Kottelat 2022). It was first described as *Exostoma andersonii* by Day (1870) from Hotham and Ponsee in Yunnan Province, China. Norman (1925) later established the genus *Glaridoglanis* and referred *Exostoma andersonii* to this genus as the type species. For decades thereafter, *G. andersonii* remained the sole recognized species, recorded from the Irrawaddy and the Yarlung Tsangpo–Brahmaputra drainages (Wu and Wu 1991; Chu and Mo 1999), until *G. ramosa* was described (Ng and Kottelat 2022). In a recent ichthyological survey, we carefully examined specimens of *Glaridoglanis* collected from the Zayul River. Comprehensive comparisons with topotypic material of *G. andersonii* based on both morphology and molecular phylogeny confirmed that these specimens belong to a distinct species. Furthermore, as these specimens could not be assigned to another valid species of this genus (*G. ramosa*), we describe them here as a new species.

## ﻿Material and methods

**Figure 1.**
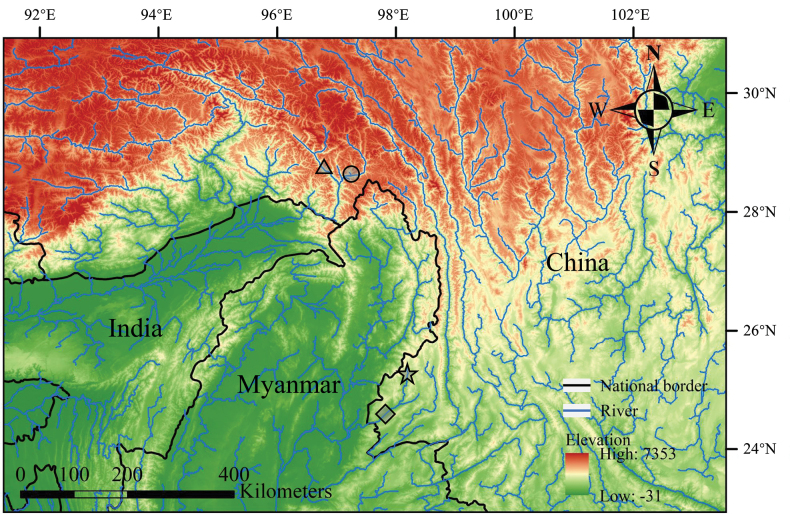
The type locality (circle) and paratype locality (triangle) of *Glaridoglanis verruciloba* sp. nov. in Xizang Autonomous Region, as well as the type locality (diamond) and the locality of type-drainage specimens (star) of *G. andersonii* in Yunnan Province, China.

Specimens of *Glaridoglanis verruciloba* sp. nov. were collected in April 2025 from mountain streams of the Sangqu River and the Gongrigabu River, two branches of the upper Zayul River. The type-drainage specimens of *G. andersonii* were collected in July 2025 from the Binglang River, the eastern branch of the Daying River, a tributary of the upper Irrawaddy River in Yunnan Province, which is approximately 100 km north-east of the type locality of the *G. andersonii* (Fig. 1). Immediately after anesthetizing the fish specimens, a small portion of pectoral-fin tissue was clipped and preserved in ethanol for subsequent DNA extraction, while whole specimens were fixed in 10% formalin and later transferred to 70% ethanol for long-term storage.

Morphological measurements were taken from the left side of the preserved specimens whenever possible using digital callipers to the nearest 0.1 mm, by a single operator. Measurements, counts and terminology methods followed Ng and Kottelat (2022). Standard length (SL) was used as the reference for body proportions, and head subunits were expressed as percentages of head length (HL). Meristic data were presented with frequencies in parentheses; when multiple values were observed, the holotype condition was marked with an asterisk. All voucher specimens were deposited at the museum of Institute of Plateau Biology of Xizang Autonomous Region (**IPBX**), Lhasa, China.

Genomic DNA was extracted from ethanol-preserved pectoral-fin tissues using a modified salt-extraction protocol (Tang et al. 2009). The mitochondrial cytochrome b (cyt b) gene was amplified by PCR with primers L14724 and H15915 (Xiao et al. 2001). Amplifications were performed in 30 µL reactions following Gong et al. (2023). PCR procedures included an initial denaturation at 94 °C for 3 min, followed by 35 cycles of denaturation at 94 °C for 30 s, annealing at 56 °C for 30 s, and extension at 72 °C for 1 min, with a final extension at 72 °C for 10 min. The amplified products were purified and sequenced bidirectionally by Tianyi Huayu Biotech Co., Ltd (Wuhan, China).

Phylogenetic analyses were performed based on 12 newly obtained cyt b sequences of *G. verruciloba* sp. nov. and *G. andersonii*, together with 26 sequences retrieved from GenBank representing additional glyptosternine taxa (Table 1). *Glyptothorax minimaculatus* and *G. zanaensis* were included as the outgroup taxa.

**Table 1.** List of species, distributional drainages, and GenBank accession numbers (cytochrome b) used in the phylogenetic analyses.

Species	Distributional drainage	GenBank accession no.
*Chimarrichthys kishinouyei*	Yangtze River	AY207478
*Chimarrichthys longus*	Red River	DQ192485
*Creteuchiloglanis gongshanensis*	Nujiang–Salween River	NC_028516
*Creteuchiloglanis kamengensis*	Yarlung Tsangpo–Brahmaputra River	NC_045213
*Creteuchiloglanis macropterus*	Nujiang–Salween River	NC_028509
*Exostoma gaoligongense*	Nujiang–Salween River	NC_056351
*Exostoma tenuicaudatum*	Yarlung Tsangpo–Brahmaputra River	NC_065343
*Exostoma tibetanum*	Yarlung Tsangpo–Brahmaputra River	NC_065342
*Glaridoglanis andersonii* Hap1	Irrawaddy River	PX508675
*Glaridoglanis verruciloba* Hap1	Yarlung Tsangpo–Brahmaputra River	PX508676
*Glaridoglanis verruciloba* Hap2	Yarlung Tsangpo–Brahmaputra River	PX508677
*Glyptosternon maculatum*	Yarlung Tsangpo–Brahmaputra River	NC_021597
*Oreoglanis immaculata*	Nujiang–Salween River	NC_028511
*Oreoglanis macroptera*	Irrawaddy River	NC_021607
*Parachiloglanis benjii*	Yarlung Tsangpo–Brahmaputra River	MG001360
*Parachiloglanis bhutanensis*	Yarlung Tsangpo–Brahmaputra River	MG001359
*Parachiloglanis dangmechhuensis*	Yarlung Tsangpo–Brahmaputra River	MG001353
*Parachiloglanis drukyulensis*	Yarlung Tsangpo–Brahmaputra River	MG001357
*Parachiloglanis immaculata*	Yarlung Tsangpo–Brahmaputra River	OQ437239
*Pseudecheneis immaculata*	Lancang–Mekong River	MN082047
*Pseudecheneis paviei*	Red River	NC_086847
*Pseudecheneis sirenica*	Yarlung Tsangpo–Brahmaputra River	NC_021605
*Pseudexostoma brachysoma*	Nujiang-Salween River	KU987338
*Pseudexostoma yunnanense*	Irrawaddy River	NC_021604
*Tremeuchiloglanis anteanalis*	Yangtze River	NC_028513
*Tremeuchiloglanis hupingshanensis*	Yangtze River	KU356571
*Tremeuchiloglanis macrotrema*	Red River	OM428185
*Tremeuchiloglanis posteranalis*	Pearl River	OM428179
*Tremeuchiloglanis rhabdura*	Red River	OM428184
*Glyptothorax minimaculatus*	Irrawaddy River	HQ322535
*Glyptothorax zanaensis*	Nujiang-Salween River	NC_029709

The raw sequences were aligned and edited manually in ClustalX (Thompson et al. 1997). Pairwise genetic distances based on the Kimura-2-parameter (K2P) model were calculated using MEGA 7 (Kumar et al. 2016). Phylogenetic relationships were reconstructed using both Bayesian inference (BI) and maximum-likelihood (ML) methods. The best-fitting nucleotide substitution model was selected with jModelTest 2 (Darriba et al. 2012) under the corrected Akaike information criterion. The BI tree was generated using MrBayes 3 (Huelsenbeck and Ronquist 2001) under GTR + I + G model, with four simultaneous Markov chains run for 1 million generations and trees sampled every 100 generations. After discarding the first 5,000 trees as burn-in, a consensus tree was generated from the remaining 5,001 trees, and posterior probabilities were determined. The ML tree was reconstructed in RAxML 8 (Stamatakis 2014) under the GTR + I + G model, and nodal support was estimated with 1,000 bootstrap replicates.

## ﻿Taxonomy

### ﻿ Glaridoglanis verruciloba

Taxon classificationAnimaliaSiluriformesSisoridae

Gong sp. nov.

DB33DC45-6393-51F6-85AA-E8079AE7AB32

https://zoobank.org/CBAAE243-78F7-4A14-9ECA-9E2F43AF2A5D

Fig. 2, Table 2

#### Chresonymy.

*Glaridoglanis andersonii* (non Day 1870): Wu et al. 1981 (Zayul River, Zayul County, China); Wu and Wu 1991 (Zayul River, Zayul County, China); Zhang et al. 1995 (Zayul River, Zayul County, China); Chu and Mo 1999 (partim, Zayul River, Zayul County, China).

**Figure 2.**
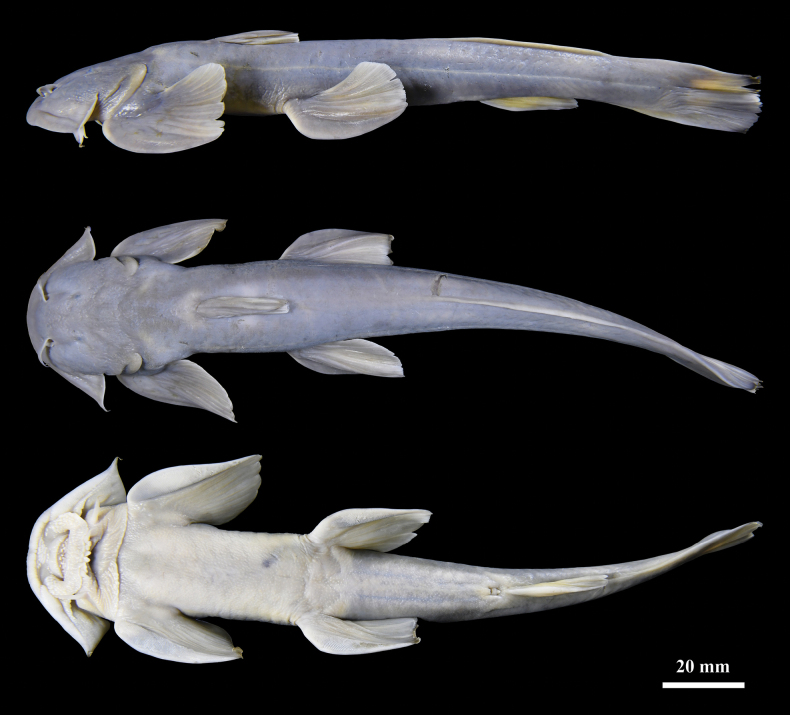
Dorsal, lateral, and ventral views of *Glaridoglanis verruciloba*, holotype, IPBX F202504001, 147.8 mm SL.

#### Type material.

***Holotype*** • IPBX F202504001, 147.8 mm SL; China, Xizang Autonomous Region, Zayul County, mountain stream flowing into Sangqu River, eastern branch of upper Zayul River; 28°35'39.31"N, 97°9'20.93"E; 1736 m a.s.l.; Zheng Gong and Yanchao Liu leg.; April 2025. ***Paratypes*** • IPBX F202504002, 1, 156.6 mm SL; data as for holotype • IPBX F202504003–F202504006, 4; 106.7–149.8 mm SL; China, Xizang Autonomous Region, Zayul County, mountain stream flowing into Gongrigabu River, western branch of upper Zayul River; 28°54'3.06"N, 96°39'54.58"E; 2016 m a.s.l.; Zheng Gong and Yanchao Liu leg.; April 2025.

#### Diagnosis.

*Glaridoglanis verruciloba* sp. nov. can be distinguished from its two congeneric species by the following combination of characters: enlarged lower lip bearing 4–7 verruciform lobes on the central-posterior margin; anus close to the anal-fin origin; ventral surface at the base of maxillary barbels densely covered with striae; 11 branched pectoral-fin rays; 5–6 branched anal-fin rays; head relatively short (HL 19.1–21.7% of SL); and mandibular barbels relatively short (inner mandibular barbel length 13.9–17.7% and outer mandibular barbel length 29.1–36.4% of HL).

#### Description.

Morphometric data as in Table 2. Head and body greatly depressed. Dorsal profile rising gently and evenly from orbital margin to dorsal-fin origin, then sloping gradually to caudal peduncle; ventral profile nearly flat to the anal-fin base, then slightly ascending to caudal peduncle. Body depth at dorsal-fin origin 11.2–13.4% of SL, at anus 8.4–11.8% of SL. Caudal-peduncle depth 6.9–9.7% of SL. Head moderate in size. Snout rounded and flattened, 49.6–53.9% of HL. Rostral cap with a shallow groove; groove margins papillate. Eye small, located dorsolaterally, subcutaneous. Nostrils paired, located closer to the snout tip than to eye, separated by the nasal barbels. Barbels in four pairs. Nasal barbels moderately long, extending beyond posterior orbital margin. Maxillary barbels slightly flattened, ventral surface at the base densely covered with striae, connected to the lower lip by a skin flap, free only at short distal end; tip pointed, almost reaching the base of first pectoral-fin ray. Inner mandibular barbels short and slightly flattened. Outer mandibular barbels lateral to inner pair, slightly flattened, not reaching the base of first pectoral-fin ray.

**Table 2.** Morphometric data for *Glaridoglanis verruciloba* sp. nov. and its closest congener, *G. andersonii*. *N* = sample size, SD = standard deviation.

	*Glaridoglanis verruciloba* sp. nov. (*N* = 6)			*G. andersonii* (*N* = 6)	
	Holotype	Range	Mean ± SD	Range	Mean ± SD
Standard length (mm)	147.8	106.7–156.6	138.4±17.3	114.6–184.8	154.2±23.9
**Percent of standard length**					
Body depth at dorsal-fin origin	11.8	11.2–13.4	12.4±0.8	9.8–11.8	10.5±0.7
Body depth at anus	9.0	8.4–11.8	9.7±1.1	8.5–9.9	9.2±0.5
Head length	19.1	19.1–21.7	20.8±0.9	22.2–24.2	22.8±0.4
Head depth	9.7	9.6–10.3	10.0±0.2	7.2–8.5	7.9±0.4
Head width	18.1	17.4–21.1	18.9±1.2	19.2–20.6	19.9±0.5
Predorsal length	29.1	28.8–30.5	29.3±0.6	31.2–32.6	31.9±0.5
Prepectoral length	12.8	12.8–16.7	14.9±1.3	14.9–16.5	15.8±0.5
Prepelvic length	40.4	40.4–42.4	41.3±0.6	42.3–46.5	44.9±1.5
Preanal length	72.0	70.5–74.0	72.1±1.4	72.7–74.4	73.5±0.6
Preadipose length	64.6	62.4–68.1	64.6±1.8	67.1–71.3	69.7±1.5
Dorsal-fin length	12.7	11.3–14.8	13.0±1.1	13.3–16.1	14.0±1.1
Pectoral-fin length	20.8	19.8–23.1	21.3±1.3	19.4–21.6	20.4±0.8
Pelvic-fin length	19.5	17.2–19.8	18.8±1.2	16.3–18.7	17.2±0.8
Anal-fin length	15.4	13.4–15.4	14.3±0.7	14.2–16.8	15.5±0.9
Caudal-fin length	11.8	11.4–15.2	13.4±1.3	10.1–14.6	12.3±1.5
Dorsal-fin base length	6.8	6.3–8.0	7.2±0.6	6.5–7.6	7.2±0.4
Pectoral-fin base length	9.7	8.9–9.9	9.4±0.4	8.6–9.9	9.2±0.5
Pelvic-fin base length	6.6	6.1–7.1	6.5±0.3	5.4–6.1	5.8±0.3
Anal-fin base length	8.1	7.4–8.8	7.9±0.5	7.8–9.3	8.6±0.6
Adipose-fin base length	28.1	24.8–29.5	27.6±1.7	23.3–29.9	26.1±2.4
Dorsal-to-adipose distance	28.1	23.6–30.1	27.6±2.1	26.5–34.3	30.7±2.5
Pectoral–pelvic distance	17.2	15.9–19.5	17.2±1.2	20.0–23.9	21.8±1.5
Pelvic to anal distance	24.0	21.6–25.1	23.4±1.5	21.2–23.7	22.5±0.9
Vent-anal-fin origin distance	2.8	1.7–2.8	2.3±0.3	2.7–4.1	3.2±0.5
Caudal-peduncle length	19.0	16.1–20.9	18.8±1.4	16.4–19.6	17.9±1.1
Caudal-peduncle depth	7.0	6.9–9.7	7.6±0.9	7.4–8.7	7.8±0.5
**Percent of head length**					
Snout length	53.9	49.6–53.9	52.1±1.7	43.8–52.1	49.5±3.1
Mouth width	36.5	31.5–36.5	34.5±1.7	31.3–34.2	33.1±1.1
Interorbital distance	28.4	22.3–31.0	26.7±2.8	24.2–26.8	26.0±1.0
Eye diameter	5.3	4.8–7.3	6.0±0.8	4.5–6.0	5.3±0.6
Nasal barbel length	27.3	24.1–37.4	28.5±5.5	32.5–40.5	37.1±3.1
Maxillary barbel length	75.5	71.8–90.1	78.3±6.0	77.9–98.8	87.7±7.6
Inner mandibular barbel length	17.7	13.9–17.7	16.2±1.5	17.2–20.9	19.0±1.5
Outer mandibular barbel length	33.3	29.1–36.4	32.2±2.4	34.2–39.7	37.4±2.3

Mouth inferior, gape width 31.5–36.5% of HL. Lips thick, fleshy, and papillate. Upper lip covered with tiny papillae; lower lip enlarged, with anastomosing rounded plaques, bearing 4–7 irregular verruciform lobes on the central-posterior margin. Postlabial groove interrupted. Mental region with a prominent median depression. Teeth embedded in skin, short, robust and chisel-shaped; similar in form on both jaws, but arranged in a single crescentic band on upper jaw and two well-separated triangular patches on lower jaw. Palate edentulous. Gill opening narrow, extending from the base of the first pectoral-fin ray to a position anterior and dorsal to the last pectoral-fin ray; posterior margin of the branchiostegal membrane forming a distinct boundary between cephalic and thoracic regions.

Dorsal fin without spine, with i,5 (1) or i,6 (5*) rays. Adipose fin with long base; anterior extremity at approximately midway between bases of pelvic and anal fins; posterior extremity separate from upper procurrent caudal-fin rays without incision. Pectoral fin enlarged and ovoid when expanded, with i,11 (6) rays; margin slightly concave; the first unbranched ray flattened, ventral surface with closely-arranged striae. Pelvic fin enlarged and ovoid when expanded, with i,5 (6) rays; the first unbranched ray flattened, ventral surface with closely-arranged striae; tip not reaching anus when adpressed. Anal fin with i,5 (1) or i,6 (5*) rays; posterior margin slightly concave; tip reaching approximately midway between anal-fin origin and caudal-fin base. Caudal fin nearly truncate when depressed, with i,14,i (1) or i,15,i (5*) rays. Chest and abdomen densely covered with minute papillae. Anus and urogenital openings located near the anal-fin origin.

#### Colouration.

In life, dorsal and lateral surfaces yellow brown; ventral surface pale pink; ventral surface of the first rays of pectoral and pelvic fins faintly pink; all fins with yellowish distal margins. In 70% ethanol, head and dorsum pale gray, ventral region dark yellow; dorsal, anal, and caudal fins yellowish gray; dorsal surfaces of pectoral and pelvic fins yellowish gray, ventral surfaces dark yellow.

#### Distribution and habitat.

**Figure 3.**
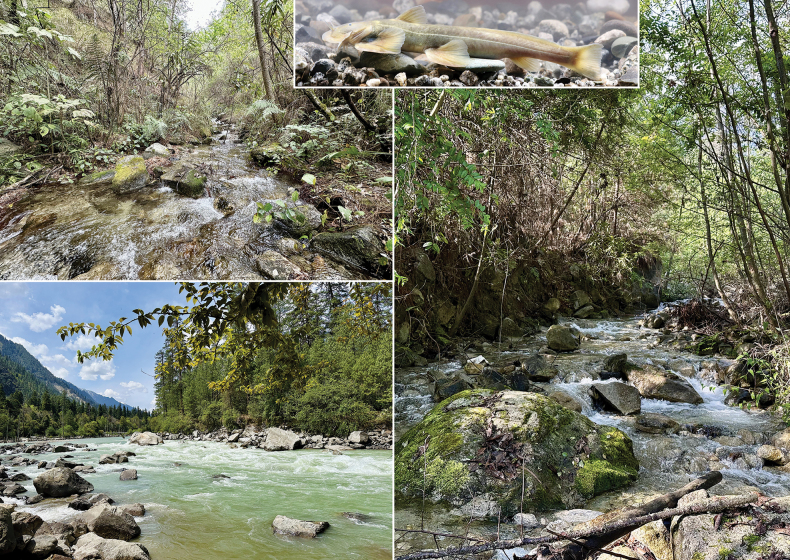
Type locality of *Glaridoglanis verruciloba* sp. nov.: a mountain stream flowing into the Sangqu River, the eastern branch of the upper Zayul River (upper left); paratype locality: a mountain stream flowing into the Gongrigabu River, the western branch of the upper Zayul River (right); the mainstem of Gongrigabu River (lower left); and a living specimen (approx. 140 mm SL) photographed in an aquarium.

This species is presently known only from the Zayul River (= upper Lohit River) drainage, a tributary of the Brahmaputra River, south-eastern Tibet, China. It occurs mainly in mountain streams and is less frequently found in the river mainstem (Fig. 3). At the type locality during sampling period, the water temperature was 4.1–7.5 °C, the dissolved oxygen was 9.46–12.58 mg/L, and the pH was 7.86–8.32. Sympatric fishes mainly included *Creteuchiloglanis kamengensis* (Jayaram, 1966) and *Schizothorax molesworthi* Tsao, 1964.

#### Etymology.

The specific epithet is derived from the Latin *verruca* (= wart) and *lobus* (= lobe), referring to the verruciform lobes on the central-posterior margin of lower lip. As *Glaridoglanis* is feminine (fide Kottelat 2013), the specific epithet is treated accordingly. The Chinese common name of this species is 疣叶凿齿鮡 (Yóu yè záo chǐ zhào), which literally means “wart-lobed chiseled-tooth catfish”.

#### Molecular phylogenetic analysis.

**Figure 4.**
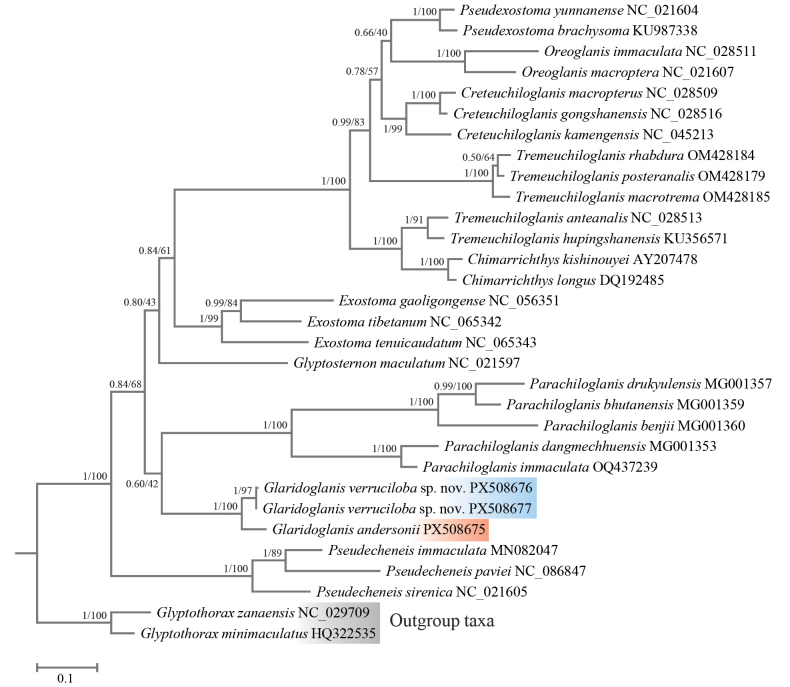
Phylogenetic tree of 28 glyptosternine catfishes inferred from mitochondrial cytochrome b gene sequences. Numbers on nodes before and after slash (/) represent posterior probabilities of Bayesian inference method and bootstrap values of maximum-likelihood method, respectively.

After alignment, 1138 bps of cyt b gene sequences were obtained from 28 species of the subfamily Glyptosterninae and two outgroup taxa for molecular phylogenetic analyses. Among these sites, 492 were variable and 463 were parsimony-informative. Within the genus *Glaridoglanis*, two haplotypes were detected from six individuals of *G. verruciloba* sp. nov., and a single haplotype was determined from six individuals of *G. andersonii*. Phylogenetic tree reconstructed based on the BI and ML methods yielded congruent topologies; therefore, only the ML tree was presented, with posterior probabilities from BI method and bootstrap values from ML method indicated at the nodes (Fig. 4). The resulting topology strongly supported the monophyly of *Glaridoglanis*, with each of its two species forming well-supported clade. Further, the genus *Glaridoglanis*, together with *Parachiloglanis*, was resolved as a relatively basal lineage within the glyptosternine catfishes, although this relationship was weakly supported. Pairwise genetic distance based on the K2P model demonstrated a 3.1% sequence divergence between the two *Glaridoglanis* species, while divergences between *G. verruciloba* and other glyptosternine catfishes were summarized in Suppl. material 1.

## ﻿Discussion

Until now, only two valid species of *Glaridoglanis* have been formally recognized, both originally described from the Irrawaddy drainage (Ng and Kottelat 2022). *Glaridoglanis andersonii* was first recognized by Day (1870) based on the specimens collected from Hotham (= Husa Township, 24°28'09"N, 97°53'54"E) and Ponsee (= Bangxi, approximately 24°28'N, 97°42'E) in Yingjiang County of Yunnan Province, which is within to the upper Irrawaddy drainage (Ng and Kottelat 2022). In the original description of *G. andersonii*, which had been assigned to *Exostoma*, the diagnosis is rather brief and lacks several crucial morphological characters now regarded as diagnostic within *Glaridoglanis*. As a result, for a long time, specimens from adjacent river drainages, including the Zayul River, were referred to *G. andersonii* due to the absence of comprehensive comparative material across localities (see Chresonymy above).

**Figure 5.**
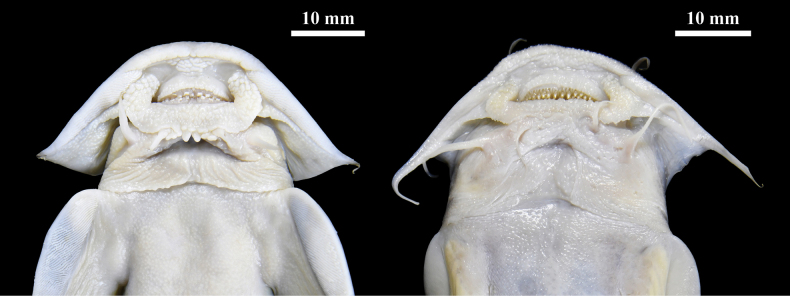
Ventral view of mouth structures of *Glaridoglanis verruciloba* sp. nov. (left, holotype, 147.8 mm SL) and *G. andersonii* (right, uncatalogued specimen, 146.5 mm SL).

Our results clearly demonstrate that *G. verruciloba* can be distinguished from *G. andersonii* by the following morphological characters: presence of verruciform lobes on the central-posterior margin of lower lip (vs absence; Fig. 5), presence of dense striae on ventral surface at the base of maxillary barbels (vs absence), a shorter head (19.1–21.7% of SL vs 22.2–24.2%), and shorter mandibular barbels (inner mandibular barbel length 13.9–17.7% of HL and 3.0–3.7% of SL vs 17.2–20.9% and 3.9–4.8%; outer mandibular barbel length 29.1–36.4% of HL and 6.1–7.4% of SL vs 34.2–39.7% and 7.8–9.1%). In addition, *G. verruciloba* can be readily differentiated from *G. ramosa* by the more posterior position of anus, which is close to the origin of anal fin (vs just anterior to the posterior margin of adpressed pelvic fin; see Ng and Kottelat 2022: fig. 1), presence of verruciform lobes on the central-posterior margin of lower lip (vs absence), fewer branched pectoral-fin rays (11 vs 13–14), more branched anal-fin rays (5–6 vs 4), and shorter mandibular barbels (inner mandibular barbel length 13.9–17.7% of HL vs 21–26%; outer mandibular barbel length 29.1–36.4% of HL vs 32–49%).

The validity of this new species is further corroborated by molecular phylogenetic evidence. Analyses inferred from cyt b gene sequences reveal that *G. verruciloba* exhibits substantial genetic divergence from its closest congener, *G. andersonii*. Such level of divergence is consistent with interspecific discrimination commonly recognized in most teleosts (Liu et al. 2022; Chen et al. 2025). Moreover, phylogenetic reconstruction placed all examined individuals of *G. verruciloba* in a well-supported monophyletic clade, which is sister to *G. andersonii*. Subsequently, the genus *Glaridoglanis* clustered with *Parachiloglanis* near the basal position within the glyptosternine phylogeny. Together, these morphological and molecular data provide robust evidence for the recognition of the Zayul River specimens as a distinct species.

From a conservation perspective, *G. verruciloba* appears to be highly vulnerable due to its narrow geographical distribution, being restricted solely to the Zayul River. However, Kadu and Das (2021) reported *G. andersonii* from the Mabung Stream in the Zangnan (Southern Tibet) region of China (referred to as “Arunachal Pradesh” in Indian sources). Their specimen exhibited indistinct verruciform lobes on the central-posterior margin of lower lip (Kadu and Das 2021: fig. 3a), a diagnostic character of *G. verruciloba*. If correctly identified, this record would extend the known range of this species westward into the Yarlung Tsangpo–Brahmaputra drainage, thereby slightly reducing its apparent endemism. In addition, the indigenous Dengba people have traditionally depended on fishing for subsistence. Our recent ichthyological surveys indicated that local fish populations had experienced a pronounced decline, most likely driven by intensive and largely unregulated fishing. Furthermore, the increasing occurrence of invasive fish species—possibly introduced through aquaculture practices or accidental releases—has disrupted the native community structure and may further exacerbate competition for limited resources. During our recent surveys of the Zayul River mainstem, we recorded some invasive species, including *Carassius auratus* (Linnaeus, 1758), *Misgurnus anguillicaudatus* (Cantor, 1842), *Silurus asotus* Linnaeus, 1758, and other species. Given these threats, the long-term persistence of *G. verruciloba* appears uncertain without prompt conservation intervention. Therefore, we recommend implementing strict fishing bans within critical habitats, establishing protected zones along mountain streams, and actively controlling invasive species as to ensure the survival of this endemic torrent catfish.

### ﻿Comparative material

*Glaridoglanis andersonii*: uncatalogued specimens deposited at the Zoological Collection, College of Life Sciences, Zaozhuang University, 114.6–184.8 mm SL, the Binglang River, a secondary tributary of the upper Irrawaddy River, Tengchong County in Yunnan Province, China.

*Glaridoglanis ramosa*: data from Ng and Kottelat (2022).

## Supplementary Material

XML Treatment for Glaridoglanis verruciloba

## ﻿Additional information

### Use of AI

No use of AI was reported.

### Author ORCIDs

Zheng Gong https://orcid.org/0000-0002-0176-696X

Huanshan Wang https://orcid.org/0000-0003-0542-5588

Yanchao Liu https://orcid.org/0000-0002-2530-8787

### Data availability

All of the data that support the findings of this study are available in the main text or Supplementary Information.

## Supplementary materials

Supplementary material 1 Kimura 2-parameter genetic distances (%, below the diagonal) and standard errors (%, above the diagonal) among 28 glyptosternine catfishes derived from mitochondrial cytochrome b gene sequences. Data type docx https://binary.pensoft.net/file/1489069This dataset is made available under the Open Database License (http://opendatacommons.org/licenses/odbl/1.0/). The Open Database License (ODbL) is a license agreement intended to allow users to freely share, modify, and use this Dataset while maintaining this same freedom for others, provided that the original source and author(s) are credited.Zheng Gong, Huanshan Wang, Yanchao Liu, Jianchuan Li
