# Supplementary material for: ﻿Glaridoglanis verruciloba sp. nov., a new glyptosternine catfish (Siluriformes, Sisoridae) from the Zayul River in southeastern Tibet, China
Source: Zookeys. 2025 Dec 10;1262:289–301. doi: 10.3897/zookeys.1262.172819 (PMC12712639; doi:10.3897/zookeys.1262.172819)
Supplement: Supplementary material 1 — Kimura 2-parameter genetic distances (%, below the diagonal) and standard errors (%, above the diagonal) among 28 glyptosternine catfishes derived from mitochondrial cytochrome b gene sequences. [file zookeys-1262-289_article-172819__-s001.docx]

**Supplementary material 1** Kimura-2-Parameter genetic distances (%, below the diagonal) and standard errors (%, above the diagonal) among 28 glyptosternine catfishes derived from mitochondrial cytochrome *b* gene sequences.

|  | **1** | **2** | **3** | **4** | **5** | **6** | **7** | **8** | **9** | **10** | **11** | **12** | **13** | **14** | **15** | **16** | **17** | **18** | **19** | **20** | **21** | **22** | **23** | **24** | **25** | **26** | **27** | **28** |
| --- | --- | --- | --- | --- | --- | --- | --- | --- | --- | --- | --- | --- | --- | --- | --- | --- | --- | --- | --- | --- | --- | --- | --- | --- | --- | --- | --- | --- |
| **1. *Glaridoglanis verruciloba* sp. nov.** |  | 0.5 | 1.6 | 1.6 | 1.6 | 1.6 | 1.6 | 1.4 | 1.3 | 1.2 | 1.2 | 1.6 | 1.6 | 1.5 | 1.5 | 1.5 | 1.5 | 1.4 | 1.5 | 1.6 | 1.5 | 1.6 | 1.5 | 1.6 | 1.6 | 1.6 | 1.7 | 1.6 |
| **2. *Glaridoglanis andersonii*** | 3.1 |  | 1.5 | 1.6 | 1.6 | 1.6 | 1.6 | 1.2 | 1.3 | 1.4 | 1.3 | 1.7 | 1.7 | 1.6 | 1.5 | 1.6 | 1.5 | 1.5 | 1.4 | 1.5 | 1.4 | 1.6 | 1.5 | 1.6 | 1.5 | 1.5 | 1.5 | 1.5 |
| **3. *Chimarrichthys kishinouyei*** | 20.1 | 19.6 |  | 0.4 | 1.2 | 1.2 | 1.2 | 1.5 | 1.5 | 1.5 | 1.5 | 1.3 | 1.4 | 1.9 | 2.0 | 1.9 | 2.0 | 1.9 | 1.7 | 1.7 | 1.6 | 1.2 | 1.2 | 0.9 | 0.9 | 1.4 | 1.3 | 1.4 |
| **4. *Chimarrichthys longus*** | 20.5 | 20.3 | 1.6 |  | 1.2 | 1.1 | 1.2 | 1.5 | 1.5 | 1.6 | 1.5 | 1.3 | 1.4 | 1.9 | 2.0 | 1.9 | 2.0 | 1.9 | 1.7 | 1.7 | 1.6 | 1.1 | 1.1 | 0.8 | 0.9 | 1.4 | 1.3 | 1.3 |
| **5. *Creteuchiloglanis gongshanensis*** | 22.6 | 22.1 | 13.3 | 12.3 |  | 0.7 | 0.4 | 1.6 | 1.5 | 1.6 | 1.6 | 1.2 | 1.2 | 2.0 | 2.0 | 1.9 | 1.9 | 1.8 | 1.7 | 1.9 | 1.7 | 0.9 | 0.9 | 1.2 | 1.1 | 1.2 | 1.2 | 1.2 |
| **6. *Creteuchiloglanis kamengensis*** | 20.4 | 19.2 | 13.0 | 12.5 | 6.3 |  | 0.8 | 1.5 | 1.5 | 1.5 | 1.5 | 1.3 | 1.2 | 1.9 | 2.0 | 1.8 | 1.9 | 1.7 | 1.7 | 1.7 | 1.7 | 1.0 | 1.0 | 1.1 | 1.1 | 1.2 | 1.2 | 1.2 |
| **7. *Creteuchiloglanis macropterus*** | 21.9 | 21.2 | 14.1 | 13.1 | 1.7 | 7.0 |  | 1.7 | 1.5 | 1.6 | 1.6 | 1.2 | 1.2 | 2.0 | 2.0 | 1.9 | 1.9 | 1.8 | 1.7 | 1.8 | 1.7 | 1.0 | 1.0 | 1.2 | 1.2 | 1.1 | 1.1 | 1.2 |
| **8. *Exostoma gaoligongense*** | 14.5 | 15.0 | 21.6 | 21.6 | 20.9 | 19.5 | 21.2 |  | 1.2 | 1.0 | 1.4 | 1.6 | 1.6 | 1.6 | 1.6 | 1.6 | 1.7 | 1.5 | 1.6 | 1.6 | 1.5 | 1.6 | 1.5 | 1.6 | 1.5 | 1.7 | 1.6 | 1.6 |
| **9. *Exostoma tenuicaudatum*** | 15.8 | 16.3 | 20.4 | 20.1 | 20.5 | 20.6 | 20.2 | 13.0 |  | 1.0 | 1.4 | 1.6 | 1.4 | 1.7 | 1.7 | 1.5 | 1.7 | 1.5 | 1.6 | 1.7 | 1.4 | 1.5 | 1.4 | 1.6 | 1.6 | 1.6 | 1.6 | 1.6 |
| **10. *Exostoma tibetanum*** | 14.8 | 16.6 | 21.9 | 22.2 | 21.5 | 20.0 | 21.6 | 10.3 | 10.7 |  | 1.3 | 1.6 | 1.6 | 1.6 | 1.7 | 1.5 | 1.7 | 1.4 | 1.7 | 1.6 | 1.5 | 1.5 | 1.4 | 1.6 | 1.5 | 1.7 | 1.6 | 1.6 |
| **11. *Glyptosternon maculatum*** | 14.7 | 15.2 | 20.2 | 19.7 | 21.4 | 20.1 | 22.0 | 17.0 | 16.0 | 15.9 |  | 1.6 | 1.8 | 1.6 | 1.7 | 1.5 | 1.6 | 1.4 | 1.4 | 1.5 | 1.3 | 1.6 | 1.6 | 1.5 | 1.5 | 1.7 | 1.7 | 1.7 |
| **12. *Oreoglanis immaculata*** | 23.4 | 23.5 | 16.0 | 15.7 | 14.8 | 15.3 | 15.3 | 22.9 | 21.9 | 22.9 | 22.5 |  | 0.9 | 1.9 | 2.1 | 1.8 | 2.0 | 1.8 | 1.8 | 1.8 | 1.8 | 1.1 | 1.1 | 1.3 | 1.2 | 1.4 | 1.4 | 1.4 |
| **13. *Oreoglanis macroptera*** | 22.4 | 22.1 | 15.9 | 15.2 | 13.1 | 13.4 | 13.2 | 20.9 | 20.1 | 20.4 | 23.5 | 8.5 |  | 2.0 | 1.9 | 1.7 | 1.9 | 1.7 | 1.8 | 1.8 | 1.8 | 1.1 | 1.1 | 1.3 | 1.3 | 1.2 | 1.2 | 1.3 |
| **14. *Parachiloglanis benjii*** | 22.1 | 22.5 | 27.7 | 28.0 | 29.3 | 27.9 | 29.3 | 23.9 | 23.9 | 23.8 | 21.7 | 27.7 | 29.5 |  | 1.0 | 1.4 | 1.1 | 1.4 | 1.7 | 1.5 | 1.6 | 1.8 | 1.8 | 1.8 | 1.8 | 1.8 | 1.9 | 1.9 |
| **15.** ***Parachiloglanis bhutanensis*** | 21.0 | 21.1 | 29.2 | 29.0 | 28.2 | 27.3 | 28.2 | 23.2 | 23.5 | 22.9 | 22.5 | 29.8 | 28.5 | 10.7 |  | 1.4 | 0.7 | 1.4 | 1.6 | 1.6 | 1.6 | 2.0 | 1.9 | 1.8 | 1.9 | 1.8 | 1.8 | 1.9 |
| **16. *Parachiloglanis dangmechhuensis*** | 19.5 | 19.8 | 26.7 | 26.7 | 26.8 | 24.6 | 26.5 | 22.4 | 20.0 | 21.3 | 19.4 | 27.0 | 25.2 | 18.7 | 18.4 |  | 1.5 | 0.6 | 1.7 | 1.7 | 1.7 | 1.8 | 1.8 | 1.8 | 1.8 | 1.8 | 1.8 | 1.8 |
| **17. *Parachiloglanis drukyulensis*** | 20.8 | 21.2 | 28.5 | 28.3 | 28.6 | 28.3 | 28.3 | 24.2 | 23.0 | 23.4 | 22.9 | 29.7 | 28.5 | 11.5 | 5.3 | 18.8 |  | 1.4 | 1.7 | 1.6 | 1.6 | 2.0 | 1.9 | 1.9 | 1.9 | 1.8 | 1.9 | 1.9 |
| **18. *Parachiloglanis immaculata*** | 18.0 | 19.3 | 26.2 | 26.0 | 25.2 | 23.2 | 24.8 | 20.6 | 19.4 | 19.4 | 18.4 | 26.2 | 24.7 | 19.0 | 17.9 | 4.0 | 18.4 |  | 1.6 | 1.7 | 1.6 | 1.6 | 1.7 | 1.7 | 1.7 | 1.7 | 1.7 | 1.8 |
| **19.** ***Pseudecheneis immaculata*** | 18.3 | 17.8 | 24.2 | 23.5 | 25.6 | 23.6 | 25.5 | 21.2 | 19.5 | 21.5 | 17.1 | 25.2 | 25.2 | 22.8 | 22.5 | 23.3 | 22.0 | 21.9 |  | 1.0 | 0.9 | 1.8 | 1.8 | 1.7 | 1.6 | 1.7 | 1.7 | 1.7 |
| **20. *Pseudecheneis paviei*** | 20.3 | 19.6 | 25.6 | 25.3 | 27.5 | 25.5 | 27.1 | 21.9 | 20.6 | 21.0 | 19.1 | 26.6 | 25.2 | 20.7 | 21.0 | 23.5 | 21.3 | 21.8 | 9.1 |  | 1.0 | 1.9 | 1.8 | 1.8 | 1.7 | 1.7 | 1.7 | 1.7 |
| **21. *Pseudecheneis*** ***sirenica*** | 18.1 | 17.8 | 23.8 | 24.1 | 25.6 | 25.2 | 25.7 | 20.4 | 18.7 | 19.4 | 17.1 | 26.7 | 26.7 | 22.7 | 22.1 | 23.0 | 21.7 | 21.3 | 8.7 | 11.7 |  | 1.8 | 1.7 | 1.6 | 1.6 | 1.5 | 1.6 | 1.6 |
| **22. *Pseudexostoma brachysoma*** | 21.9 | 22.1 | 13.9 | 13.1 | 9.1 | 8.9 | 9.9 | 20.8 | 20.4 | 20.1 | 21.5 | 12.8 | 12.6 | 27.7 | 28.2 | 25.5 | 28.2 | 23.9 | 26.3 | 28.3 | 27.4 |  | 0.5 | 1.1 | 1.2 | 1.3 | 1.2 | 1.3 |
| **23. *Pseudexostoma yunnanensis*** | 22.0 | 21.4 | 13.7 | 13.1 | 9.1 | 9.2 | 10.1 | 20.3 | 19.7 | 19.7 | 21.9 | 12.5 | 12.3 | 27.5 | 27.6 | 25.7 | 28.0 | 24.4 | 25.7 | 27.7 | 26.2 | 2.4 |  | 1.2 | 1.2 | 1.2 | 1.2 | 1.2 |
| **24. *Tremeuchiloglanis anteanalis*** | 21.6 | 20.8 | 7.3 | 7.0 | 12.8 | 12.6 | 13.6 | 20.9 | 21.0 | 21.9 | 19.7 | 14.5 | 14.1 | 26.6 | 26.5 | 26.1 | 27.4 | 24.6 | 24.3 | 26.1 | 24.7 | 13.1 | 13.3 |  | 0.5 | 1.2 | 1.3 | 1.2 |
| **25. *Tremeuchiloglanis hupingshanensis*** | 21.3 | 20.7 | 7.4 | 7.0 | 11.9 | 12.5 | 12.9 | 20.9 | 20.9 | 22.1 | 19.4 | 14.7 | 14.2 | 26.8 | 27.3 | 26.6 | 27.8 | 25.4 | 23.6 | 25.9 | 24.1 | 13.2 | 13.1 | 2.8 |  | 1.3 | 1.3 | 1.3 |
| **26. *Tremeuchiloglanis macrotrema*** | 22.1 | 21.7 | 17.0 | 16.6 | 13.3 | 14.4 | 12.1 | 22.2 | 21.7 | 23.7 | 24.0 | 17.3 | 14.8 | 26.7 | 26.7 | 26.2 | 27.3 | 24.5 | 24.1 | 25.3 | 23.7 | 14.0 | 13.6 | 14.8 | 14.9 |  | 0.5 | 0.5 |
| **27. *Tremeuchiloglanis posteranalis*** | 22.3 | 21.3 | 15.6 | 15.2 | 13.0 | 13.3 | 11.8 | 21.0 | 20.8 | 22.7 | 23.5 | 16.6 | 14.4 | 27.3 | 27.2 | 26.1 | 27.6 | 24.1 | 23.7 | 24.4 | 23.5 | 13.4 | 13.1 | 14.6 | 14.5 | 2.1 |  | 0.4 |
| **28. *Tremeuchiloglanis rhabdura*** | 22.0 | 21.0 | 15.4 | 15.2 | 13.3 | 14.4 | 12.1 | 21.4 | 21.3 | 23.0 | 23.9 | 16.8 | 14.9 | 27.9 | 27.5 | 26.5 | 27.6 | 24.8 | 23.9 | 24.4 | 23.3 | 13.6 | 13.5 | 14.5 | 14.4 | 2.5 | 1.4 |  |
